# Morphological Features of the Vertebrobasilar System Predict Ischemic Stroke Risk in Spontaneous Vertebral Artery Dissection

**DOI:** 10.1007/s12265-024-10534-6

**Published:** 2024-07-09

**Authors:** Jiajia Bao, Mateng Bai, Muke Zhou, Jinghuan Fang, Yanbo Li, Jian Guo, Li He

**Affiliations:** 1 Department of Neurology, West China Hospital, Sichuan University, Chengdu, China; 2 Key Laboratory of Biomechanics and Mechanobiology (Beihang University), Beijing, China; 3 Ministry of Education Beijing Advanced Innovation Center for Biomedical Engineering, Beijing, China; 4 School of Biological Science and Medical Engineering, Beihang University, Beijing, China; 5 Department of Neurology, West China Hospital, Sichuan University, Guoxue Xiang #37, Chengdu, Sichuan 610041 China

**Keywords:** Vertebral Artery Dissection, VAD, Morphological Features, Vertebrobasilar System, Vertebral Artery Ostium

## Abstract

The vertebral artery’s morphological characteristics are crucial in spontaneous vertebral artery dissection (sVAD). We aimed to investigate morphologic features related to ischemic stroke (IS) and develop a novel prediction model. Out of 126 patients, 93 were finally analyzed. We constructed 3D models and morphological analyses. Patients were randomly classified into training and validation cohorts (3:1 ratio). Variables selected by LASSO — including five morphological features and five clinical characteristics — were used to develop prediction model in the training cohort. The model exhibited a high area under the curve (AUC) of 0.944 (95%CI, 0.862–0.984), with internal validation confirming its consistency (AUC = 0.818, 95%CI, 0.597–0.948). Decision curve analysis (DCA) indicated clinical usefulness. Morphological features significantly contribute to risk stratification in sVAD patients. Our novel developed model, combining interdisciplinary parameters, is clinically useful for predicting IS risk. Further validation and in-depth research into the hemodynamics related to sVAD are necessary.

**Supplementary Information:**

The online version contains supplementary material available at 10.1007/s12265-024-10534-6.

## Introduction

Spontaneous vertebral artery dissection (sVAD) can present with localized and isolated symptoms, whereas some cases may progress to ischemia or subarachnoid hemorrhage (SAH) [1–3]. Over half of sVAD patients eventually develop ischemic stroke (IS), accounting for nearly 20% of IS in young and middle-aged adults and 3.7–4.5% of nontraumatic SAH [2, 4, 5].

Early detection of sVAD patients at high-risk for IS is critical for implementing preventive strategies. Although clinical factors such as hypertension and male gender are known to increase IS risk in sVAD [6–9], these factors alone are insufficient to predict IS occurrence in sVAD patients.

Recent evidence suggests that arterial morphological characteristics of the vertebrobasilar system (VBS) significantly affect the occurrence and development of sVAD [10–14]. Specifically, morphological features such as the hypoplastic vertebral artery (VA), increased VA tortuosity, and a smaller bending angle of the basilar artery, have been reported to closely correlate with altered hemodynamics in VBS, which may in turn influence the progression of sVAD [15, 16]. Thus, understanding these morphological features is vital for assessing stroke risk in sVAD [17].

Despite detailed studies on sVAD, the morphological characteristics of the vertebrobasilar system (VBS) in sVAD patients with and without IS, have not undergone thoroughly systematic investigation and still require comprehensive identification. Until now, there is no prediction model has been developed based on both clinical and morphological parameters to evaluate the IS risk in sVAD patients. In this study, we analyzed the morphologic features within VBS related to IS in sVAD using Mimics software. This software enables the accurate reconstruction of three-dimensional (3D) geometric models and semi-automatic measurement of arterial morphometrics [18, 19]. Building on this, we developed a prediction model integrating multiparametric morphologic data and clinical characteristics to predict IS risk for sVAD patients.

## Methods

### Study Participants

This study was conducted in compliance with the Transparent Reporting of a Multivariable Prediction Model for Individual Prognosis or Diagnosis (TRIPOD) statement [20]. From November 2015 to November 2022, we systematically screened and selected patients with unilateral sVAD at the Neurology Department of West China Hospital, Sichuan University. Each patient underwent a comprehensive neurological examination, including assessment for ischemic or hemorrhagic lesions and bilateral vertebral evaluation. Diagnoses of ischemic and hemorrhagic stroke were based on WHO criteria [21, 22]. The inclusion criteria were: (1) aged ≥ 18 years; (2) diagnosis of sVAD according to previously described criteria; (3) availability of pre-treatment head and neck CTA images in DICOM (Digital Imaging and Communications in Medicine standard) format. The exclusion criteria included: (1) hemorrhagic stroke or SAH; (2) multiple vertebral artery dissections; (3) undissected aneurysms or stenosis in vertebral arteries; (4) history of stroke (ischemic or hemorrhage) or transient ischemic attacks; (5) connective tissue or vascular disorders; (6) head or neck trauma history; (7) cervical intracranial artery malformations; (8) the time from sVAD onset to CTA screening exceeding 19 days; (9) poor quality of CTA imaging or incomplete information.

93 patients met these criteria and were divided into IS (75 patients) and non-IS (18 patients) groups. We recorded demographic and clinical data for each patient, including sex, age, body mass index (BMI), hypertension, hyperlipidemia, diabetes mellitus (DM) history, and smoking status.

### 3-Dimensional Reconstruction and Morphological Measurement

**Fig. 1:**
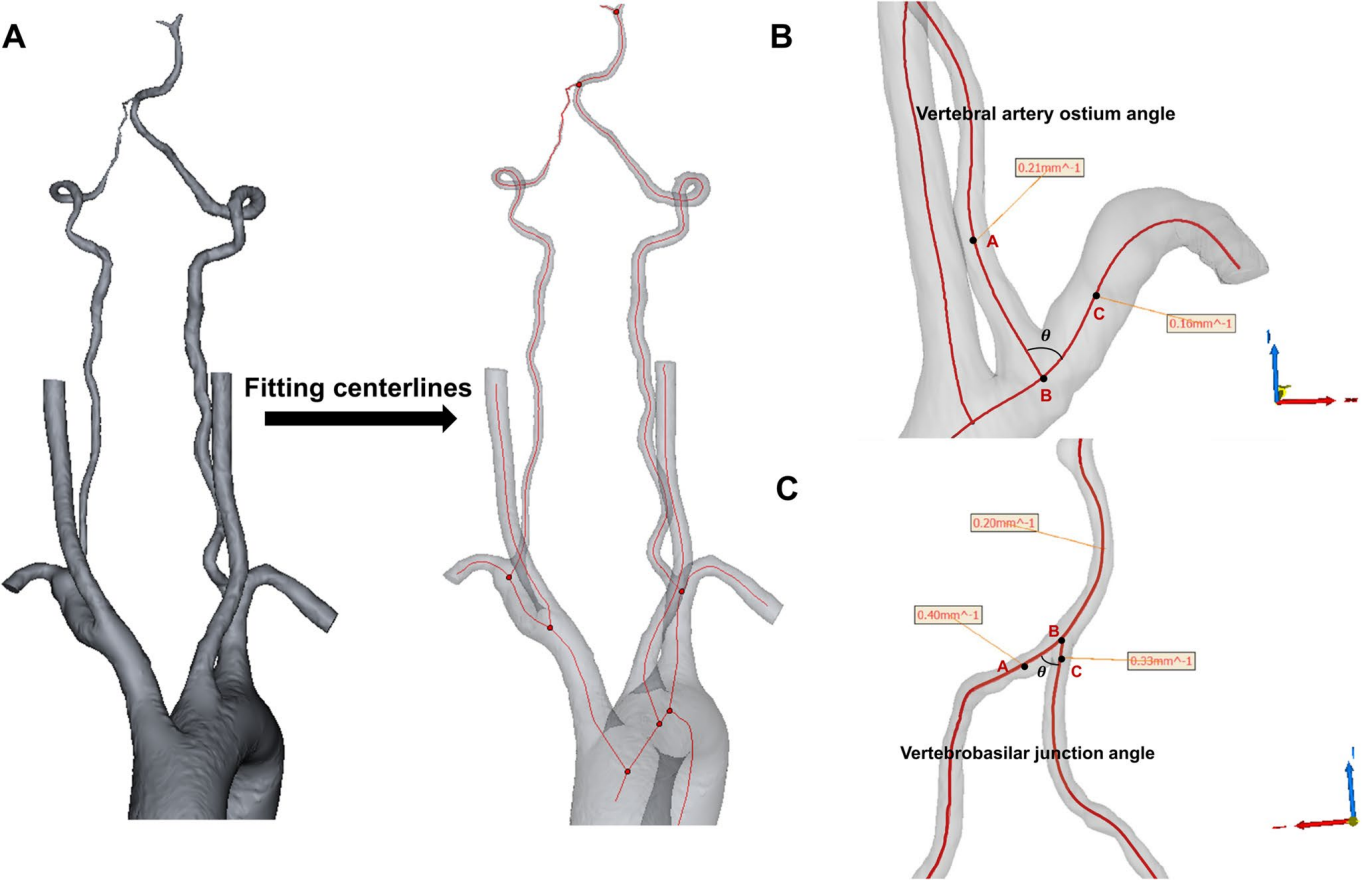
Flow chart of 3D model reconstruction and bifurcation angle measurement. **A** The reconstructed vertebrobasilar system and centerline. **B** Graphical representation of vertebral artery ostium angle assessed at the 3D model. **C** Graphical representation of vertebrobasilar junction angle assessed at the 3D model

Mimics software (version 20.0, Materialise, Leuven, Belgium) and Geomagic Studio (Geomagic, Research Triangle Park, North Carolina, USA) were utilized for creating 3-dimensional (3D) geometric models from head and neck CTA images (DICOM format). The reconstructed 3D models covered from the aortic arch to the terminate of basilar artery (BA) (Fig. 1A). Considering the posterior circulation artery is small and intricate, we further optimized these initial reconstructed 3D models into the high-quality 3D models that meet the requirements for extracting precise centerlines and delineating the most reliable vessel trajectories using Geomagic Studio. Subsequently, these high-quality 3D models were reintegrated into Mimics for extracting optimistic vessel centerlines form the catheter 3D rotational angiography (Fig. 1A).

The centerline smoothing utilized a Fourier method to eliminate high-frequency noise from the initial centerline. The centerline extraction algorithm automatically detected geometric details and set morphological parameters. The morphological parameters measured along the artery centerline included length, best-fit diameter, best-fit cross-sectional area, tortuosity, and curvature. The best-fit diameter and area were defined as the diameter and crosses-sectional area of the best-fit circle at a control point. The tortuosity = 1—(linear distance / distance along the branch). Bifurcation angles were defined using the law of cosines method in Mimics, as illustrated in Fig. 1B and C. The point A and point C were identified as locations of maximum curvature on both vessel sides forming the angle, with point B as their intersection. The coordinates of each point were recorded accordingly. The bifurcation angle was calculated using the formula:

$$\theta ={\text{cos}}^{-1}\frac{\left({x}_{1}-{x}_{2}\right)\left({x}_{3}-{x}_{2}\right)+\left({y}_{1}-{y}_{2}\right)\left({y}_{3}-{y}_{2}\right)+\left({z}_{1}-{z}_{2}\right)\left({z}_{3}-{z}_{2}\right)}{\sqrt{{\left({x}_{1}-{x}_{2}\right)}^{2}+{\left({y}_{1}-{y}_{2}\right)}^{2}+{\left({z}_{1}-{z}_{2}\right)}^{2}}\cdot \sqrt{{\left({x}_{3}-{x}_{2}\right)}^{2}+{\left({y}_{3}-{y}_{2}\right)}^{2}+{\left({z}_{3}-{z}_{2}\right)}^{2}}}$$

in which ${x}_{1}$, ${y}_{1}$, ${z}_{1}$ are the coordinates of point A, ${x}_{2}$, ${y}_{2}$, ${z}_{2}$ are the coordinates of point B, ${x}_{3}$, ${y}_{3}$, ${z}_{3}$ are the coordinates of point C.

The vertebral artery was segmented into four parts in accordance with previous criteria [23, 24]: The morphological subtypes of sVAD were classified as previously reported: (1) steno-occlusion type (steno or occlusive without aneurysmal dilatation); (2) pearl-and-string type (aneurysmal dilatation alternating with stenosis); (3) aneurysmal dilatation type (aneurysmal dilatation without stenosis) [25]. Then the measurement data along with centerline information were analyzed using the Python programming language. More detailed information is shown in Supplementary material.

### Statistical Analysis

Categorical and ordinal data were presented as percentages or frequencies, and continuous data as mean ± standard deviation (SD) or median with interquartile range (IQR). Baseline data of the IS and non-IS groups underwent univariate analysis using t-test or Mann–Whitney U-test for continuous data, and chi-square test or Fisher’s test for categorical data. Least Absolute Shrinkage and Selection Operator regression (LASSO) is utilized to select important variables through incorporating a penalty term of L1 regularization [26]. A tenfold cross-validation was applied for tuning parameter (lambda) selection in the LASSO model [27]. Additionally, we calculated the area under the receiver operating characteristic curve (AUC) of the receiver operating characteristic (ROC) with 95% confidence intervals (CI) to compare differences among morphologic parameters selected by LASSO. Sensitivity analysis involving stratified analysis and interaction test was conducted to evaluate the stoutness of these morphologic predictors across different subgroups.

All statistical analyses were performed using R and Python packages, with a statistical significance level set at *p* < 0.05, and all tests were two-sided.

### Model Development and Validation

We randomly divided the entire cohort into the training set and validation set at a 3:1 ratio. The training set was used to develop the prediction model, and the validation set was used for internal validation of the model's performance. Parameters identified by LASSO were incorporated into a logistic regression model to predict IS risk post-sVAD. The Variance Inflation Factor (VIF) served as the diagnostic index for collinearity, with a threshold below 5. The model with the smallest Akaike information criterion (AIC) was chosen as the final model, following a backward step-down selection process.

The bootstrap resampling method was used to address the class-imbalance problem in our analysis because it generates multiple datasets from the original dataset by randomly selecting observations with replacement. This method does not require assumptions about the population and ensures that each new sample is representative of the original population, allowing for accurate statistical inferences [28]. The evaluation of the performance and effect for the prediction model by internal validation in the validation cohort. The model’s discrimination capability was evaluated by the AUC of ROC with 500 bootstrap samples. The clinical applicability of the model was quantified through decision curve analysis (DCA). The relative importance score was utilized to indicate the significance of predictor variables, with the most important predictor assigned as a score of 100, while the scores of other predictors determined by their ratios to the crucial one.

## Results

### Patient Baseline Characteristics

All information for the 93 patients was complete, with no missing data. Table 1 displays the demographic characteristics of the patients. Among the 93 sVAD patients, the average age was 39 ± 10 years, and 75 patients (80.65%) developed IS. Of these patients, 35.48% (*n* = 33) were female comprised, and left VA dissection comprised 55.91% (*n* = 52) of all patients. Moreover, 58 patients had extracranial sVAD, whereas 35 patients presented with intracranial sVAD. Regarding morphology subtypes, the steno-occlusion type accounting for 49.46% of all cases, followed by the pearl-and-string type in 20.43% (*n* = 19) of patients. Nearly all patients (91.40%) had a type 1 aortic arch. There were 78 patients (83.87%) had IS. A higher proportion of male sex and extracranial dissection were observed in the IS group compared to the non-IS group (54 (72.00%) vs. 6 (33.33%), *p* = 0.002; 17 of 18 (94.44%) vs. 41 of 75 (54.67%), *p* = 0.002, respectively). However, the IS group had more patients with a history of hypertension in comparison with non-IS group (61 (81.33%) vs. 9 (50.00%), *p* = 0.006). No significant differences were found in terms of mean age, BMI, history of hyperlipidemia and DM, smoking status, morphological subtypes, vertebral artery hypoplasia (VAH) or other clinical characteristics between the IS and non-IS groups (Table 1).

**Table 1:** Study population characteristics at baseline

	Total (*n* = 93)	Non-IS (*n* = 18)	IS (*n* = 75)	*P* value
Gender, n(%)				0.002*
Female	33 (35.48%)	12 (66.67%)	21 (28.00%)	
Male	60 (64.52%)	6 (33.33%)	54 (72.00%)	
Age (years), mean ± SD	39 ± 10	41 ± 12	38 ± 9	0.251
BMI	23.56 ± 2.90	23.95 ± 2.95	23.47 ± 2.91	0.533
SBP	134.17 ± 21.06	132.67 ± 19.00	134.53 ± 21.62	0.738
DBP	86.71 ± 14.83	88.50 ± 18.13	86.28 ± 14.04	0.571
Hypertension, n(%)	70 (75.27%)	9 (50.00%)	61 (81.33%)	0.006*
Hyperlipidemia, n(%)	2 (2.15%)	1 (5.56%)	1 (1.33%)	0.267
Diabetes, n(%)	2 (2.174%)	1 (5.88%)	1 (1.33%)	0.246
Smoking, n(%)	29 (31.18%)	4 (22.22%)	25 (33.33%)	0.361
Dissection side, n(%)				0.306
Left	52 (55.91%)	12 (66.67%)	40 (53.33%)	
Right	41 (44.09%)	6 (33.33%)	35 (46.67%)	
Dissection location, n(%)				0.002*
Extracranial	58 (62.37%)	17 (94.44%)	41 (54.67%)	
Intracranial	35 (37.63%)	1 (5.56%)	34 (45.33%)	
Morphology subtypes, n(%)				0.139
Pearl-and-string appearance	19 (20.43%)	1 (5.56%)	18 (24.00%)	
Steno-occlusion only	46 (49.46%)	9 (50.00%)	37 (49.33%)	
Aneurysmal dilatation only	28 (30.11%)	8 (44.44%)	20 (26.67%)	
VAH, n(%)				0.711
None	66 (70.97%)	14 (77.78%)	52 (69.33%)	
Ipsilateral	16 (17.20%)	1 (5.56%)	7 (9.33%)	
Opposite	6 (6.45%)	3 (16.67%)	11 (14.67%)	
Bilateral	5 (5.38%)	0 (0.00%)	5 (6.67%)	
PICA occlusion, n(%)				0.371
None	31 (33.33%)	3 (16.67%)	28 (37.33%)	
Ipsilateral	31 (33.33%)	8 (44.44%)	23 (30.67%)	
Opposite	20 (21.51%)	4 (22.22%)	16 (21.33%)	
Bilateral	11 (11.83%)	3 (16.67%)	8 (10.67%)	
Types of aortic arch, n(%)				0.608
Type 1	85 (91.40%)	17 (94.44%)	68 (90.67%)	
Type 2	8 (8.60%)	1 (5.56%)	7 (9.33%)	

Values are n (%), mean ± SD, or median (quartiles). *Statistically significant at *P* < 0.05

*IS* ischemic stroke, *BMI* body mass index, *SBP* Systolic Blood Pressure, *DBP* Diastolic Blood Pressure, *VAH* vertebral artery hypoplasia, *PICA* Posterior Inferior Cerebellar Artery

### Morphologic Characterization

Table 2 presents the morphological parameters. Statistically significant differences in morphological data were observed between the IS and non-IS groups, including length, tortuosity, curvature, cross-sectional area, artery diameter, and ratio of diameters on both sides. Distinct differences were also noted in various angles including VA ostium (VAO), VA-BA, VA converge, BA angles. The sVAD patients with IS exhibited a smaller angle at the VAO (75.97° ± 14.77 vs. 84.88° ± 15.36, *p* = 0.025) and slightly higher curvature of the total dissection-side VA (0.18 ± 0.06 vs. 0.14 ± 0.02, *p* = 0.029). Additionally, there were no statistical differences between the two groups in terms of the length of the dissection area, curvature of the dissection area, tortuosity of dissection area, diameter of dissection, and cross-sectional area of dissection.

**Table 2:** Morphology characteristics of vertebrobasilar system in sVAD patients with or without IS

Parameters	Total (*n* = 93)	Non-IS (*n* = 18)	IS (*n* = 75)	OR (95%CI)	*P* value
Centerline length (cm)					
V1	49.09 ± 10.79	47.96 ± 7.49	49.36 ± 11.47	0.14 (-0.37, 0.66)	0.625
V2	67.17 ± 8.32	65.28 ± 11.03	67.62 ± 7.55	0.25 (-0.27, 0.76)	0.286
V3	68.87 ± 13.11	70.09 ± 12.29	68.58 ± 13.37	0.12 (-0.40, 0.63)	0.663
V4	49.80 ± 14.21	45.85 ± 10.22	50.75 ± 14.91	0.38 (-0.13, 0.90)	0.190
Tortuosity					
V1	0.07 ± 0.09	0.11 ± 0.14	0.06 ± 0.08	0.40 (-0.12, 0.91)	0.063
V2	0.03 ± 0.03	0.04 ± 0.03	0.03 ± 0.03	0.25 (-0.26, 0.77)	0.305
V3	0.57 ± 0.08	0.60 ± 0.09	0.57 ± 0.08	0.33 (-0.18, 0.85)	0.188
V4	0.15 ± 0.13	0.15 ± 0.11	0.14 ± 0.13	0.09 (-0.42, 0.61)	0.738
Curvature					
V1	0.17 ± 0.08	0.15 ± 0.03	0.17 ± 0.09	0.33 (-0.19, 0.85)	0.307
V2	0.16 ± 0.08	0.13 ± 0.06	0.16 ± 0.09	0.43 (-0.08, 0.95)	0.146
V3	0.18 ± 0.09	0.15 ± 0.05	0.19 ± 0.10	0.47 (-0.05, 0.99)	0.128
V4	0.19 ± 0.14	0.14 ± 0.07	0.20 ± 0.15	0.48 (-0.04, 1.00)	0.120
Artery cross-sectional area (cm^2^)					
V1	5.44 ± 3.47	5.82 ± 4.06	5.35 ± 3.34	0.13 (-0.39, 0.64)	0.602
V2	5.81 ± 4.02	6.66 ± 6.22	5.60 ± 3.31	0.21 (-0.30, 0.73)	0.317
V3	5.72 ± 3.53	6.33 ± 4.35	5.58 ± 3.32	0.20 (-0.32, 0.71)	0.417
V4	4.95 ± 3.83	4.71 ± 3.30	5.00 ± 3.96	0.08 (-0.44, 0.59)	0.775
Artery diameter (cm)					
V1	2.48 ± 0.78	2.54 ± 0.92	2.46 ± 0.75	0.09 (-0.43, 0.60)	0.729
V2	2.57 ± 0.84	2.68 ± 1.10	2.54 ± 0.77	0.15 (-0.37, 0.66)	0.529
V3	2.56 ± 0.77	2.69 ± 0.79	2.53 ± 0.77	0.19 (-0.32, 0.71)	0.458
V4	2.32 ± 0.83	2.32 ± 0.71	2.32 ± 0.85	0.01 (-0.50, 0.52)	0.972
Ratio of vertebral artery diameters on both sides					
V1	0.84 ± 0.80	0.86 ± 0.34	0.83 ± 0.88	0.04 (-0.48, 0.55)	0.912
V2	0.69 ± 0.54	0.69 ± 0.26	0.70 ± 0.59	0.02 (-0.50, 0.53)	0.954
V3	0.99 ± 0.78	1.00 ± 0.48	0.99 ± 0.83	0.01 (-0.51, 0.52)	0.98
V4	0.86 ± 0.44	0.89 ± 0.45	0.85 ± 0.45	0.10 (-0.41, 0.62)	0.692
VA-BA angle (°)	141.31 ± 19.37	141.78 ± 12.75	141.20 ± 20.71	0.03 (-0.48, 0.55)	0.91
VA ostium angle (°)	77.69 ± 15.22	84.88 ± 15.36	75.97 ± 14.77	0.59 (0.07, 1.11)	0.025*
VA converge angle (°)	62.12 ± 18.77	57.80 ± 28.42	63.16 ± 15.72	0.23 (-0.28, 0.75)	0.279
BA angle (°)	147.00 ± 15.83	143.25 ± 18.19	147.93 ± 15.19	0.28 (-0.24, 0.80)	0.264
BA tortuosity	0.07 ± 0.06	0.08 ± 0.07	0.07 ± 0.06	0.11 (-0.41, 0.62)	0.673
BA length	25.03 ± 6.77	27.36 ± 7.04	24.48 ± 6.63	0.42 (-0.10, 0.94)	0.105
Dissection parameters					
Length (cm)	46.68 ± 59.17	24.81 ± 15.57	51.92 ± 64.44	0.58 (0.06, 1.10)	0.081
Curvature	0.25 ± 0.15	0.20 ± 0.08	0.26 ± 0.16	0.49 (-0.03, 1.01)	0.113
Tortuosity	0.16 ± 0.18	0.13 ± 0.14	0.17 ± 0.19	0.25 (-0.27, 0.76)	0.391
Sectional area (cm^2^)	5.80 ± 7.86	6.84 ± 7.99	5.55 ± 7.86	0.16 (-0.35, 0.68)	0.534
Diameter (cm)	2.30 ± 1.35	2.53 ± 1.45	2.25 ± 1.33	0.21 (-0.31, 0.72)	0.422

Values are n (%), mean ± SD, or median (quartiles)

^*^Statistically significant at *P* < 0.05

*IS* ischemic stroke, *OR* odds ratio, *CI* confidence intervals, *VA* vertebral artery, *BA* basilar artery

### Variables Selection and Model Development

**Fig. 2:**
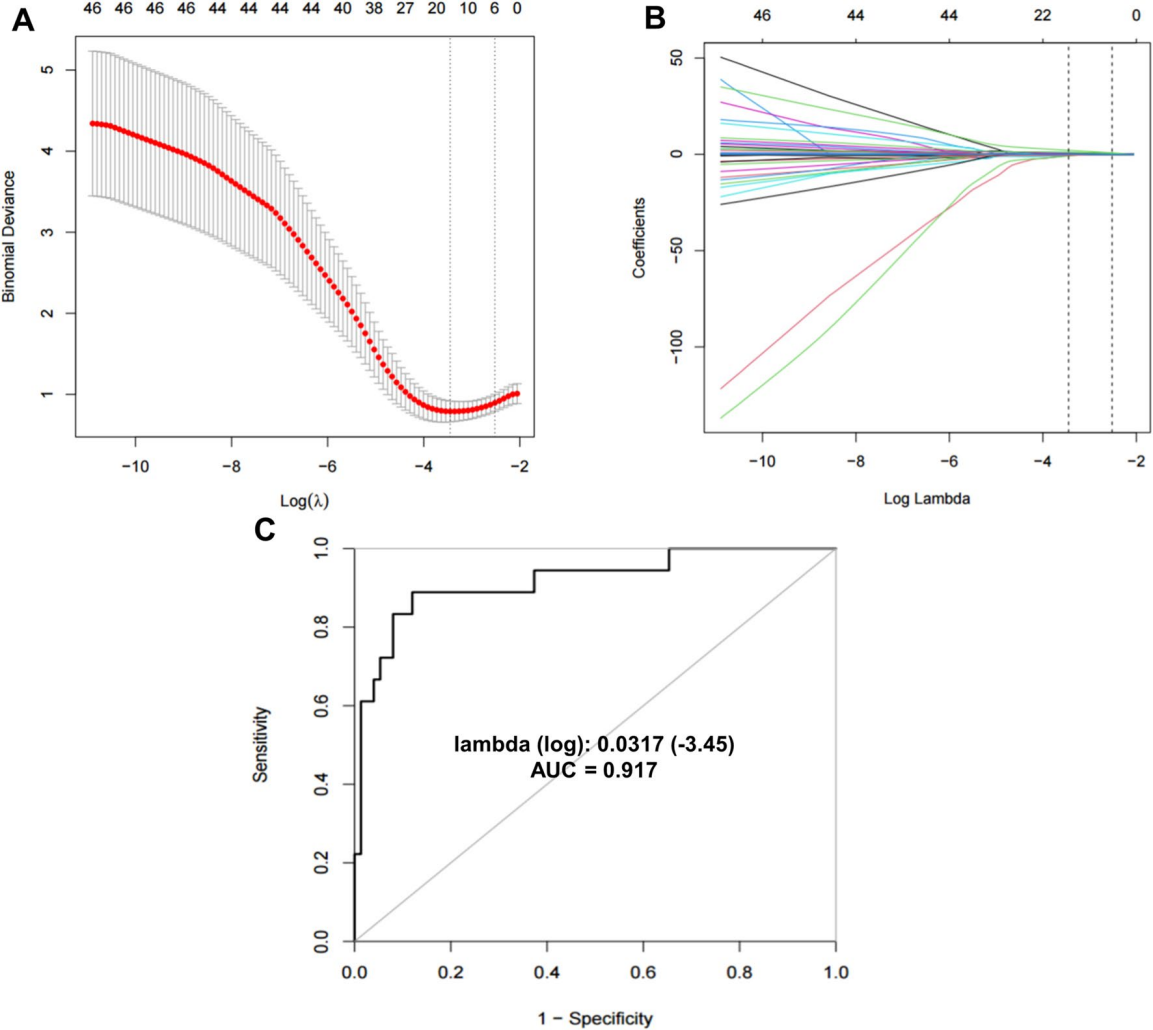
Variables selection by LASSO analysis. **A** Adjustment parameters in the LASSO model. The optimal penalization coefficient in the LASSO is screened through tenfold cross-validation. **B** LASSO coefficient diagram of potential predictors. **C** ROC curve for the LASSO model

Figure 2A illustrates the partial likelihood deviance (binomial deviance) curve, and Fig. 2B shows the coefficient profile plot. At the optimal λ value of 0.0317 (-3.45), 12 variables were chosen, including sex, history of hypertension, hyperlipidemia, DM, dissection area (extracranial or intracranial), centerline length of the BA, centerline length of the dissection, VAO angle, curvature of the V2 segment, curvature of the V3, centerline length of the V4 segment, and VAH.

Subsequently, the training cohort (71 patients (57 with IS and 14 with non-IS)) were used to develop a predictive model based on logistic regression, integrating the variables selected by the LASSO regression. The VIF was used to assess collinearity, leading to the removal of hyperlipidemia and DM given their high collinearity (VIF > 10). The final model retained ten variables, including dissection area (extracranial or intracranial), centerline length of the BA, centerline length of the V4 segment, curvature of the V2 segment, curvature of the V3, VAO angle, sex, history of hypertension, dissection length, and VAH. Notably, a narrow VSO angle, increased curvature in V2 and V3, short BA length, long V4 length, male sex, presence of hypertension, intracranial dissection, extended dissection length, and vertebral artery hypoplasia were identified as factors associated with an elevated risk of IS in sVAD patients.

### Predictive Performance and Validation

**Fig. 3:**
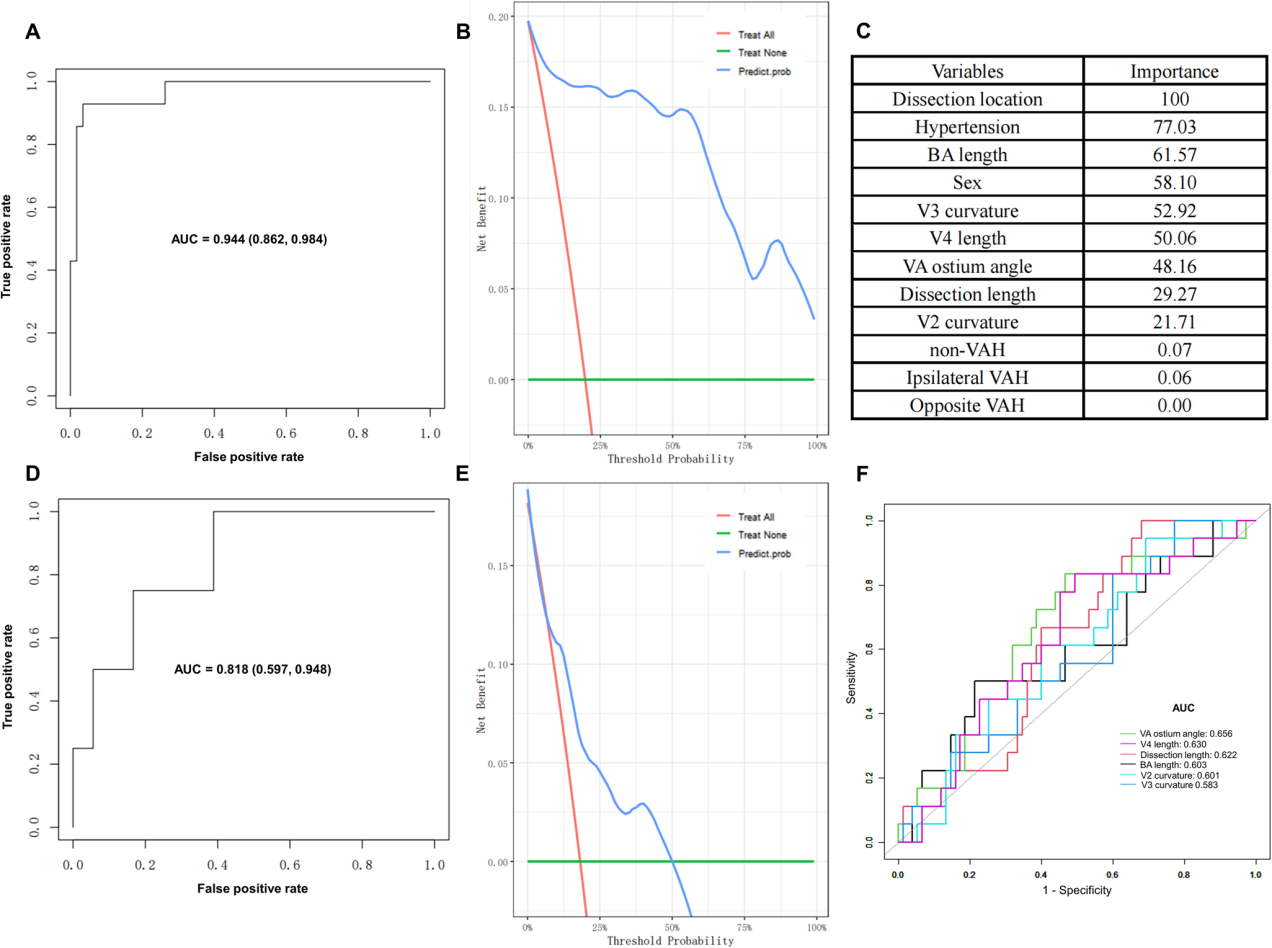
Combining morphological parameters and clinical characteristics for predicting IS in sVAD. **A** ROC curve of the prediction model in the training cohort. **B** Decision curve analysis of the prediction model in the training cohort. **C** Importance of predictors. **D** ROC curve of the prediction model in the validation cohort. **E** Decision curve analysis of the prediction model in the validation cohort. **F** Comparisons of predictive values among potentially significant morphometrics linked to IS in sVAD

The developed prediction model in the training cohort was employed to assess the risk of IS in sVAD patients. The ROC, presented in Fig. 3A, demonstrated an AUC of 0.944 (95%CI, 0.862–0.984), indicating the model’s effectiveness and accuracy. The validation cohort (22 patients (18 with IS and 4 with non-IS)) was used to internal validate the performance of the prediction model. The AUC of validation cohort displayed a good consistency of the predictive model (AUC = 0.818, 95%CI (0.597–0.948)), as shown in Fig. 3D. The DCA quantified the net benefit across various threshold probabilities, demonstrating the clinical utility of the prediction model in both the training and validation cohorts (Fig. 3B and E). Figure 3C shows the importance of predictors in assessing IS risk in sVAD patients. Among all predictors, the dissection area was the most predictive of IS risk, followed by the presence of HP. Regarding morphological features, BA length was the most important variable, with the curvature of the V3 segment being the second most significant, followed by the length of the V4 segment and the VAO angle.

### ROC and Sensitivity Analysis of Morphologic Features

We further compared the predictive power of morphologic features through ROC analysis, as shown in Fig. 3F. The VAO angle emerged as the morphologic predictor with the highest AUC (0.656, 95%CI, 0.518 to 0.775). In contrast, curvature of the V3 demonstrated the lowest AUC value (0.583, 95%CI, 0.436 to 0.708). The results of stratified analysis and interaction tests, presented in Table 3, indicated no significant interactions at the *p* < 0.05 level. This consistency of morphologic features across all examined subgroups strengthens the reliability of the model's findings. Optimal cut-off values for these morphologic predictors are detailed in the supplementary materials (Table S1).

**Table 3:** Stratification analysis of morphological features across different subgroups

	VAO angle^§^	V4 length^§^	V2 curvature^§^	V3 curvature^§^	BA length^§^
Gender					
Female	1.05 (0.98, 1.13) 0.1772	1.02(0.95, 1.10) 0.5896	0.00(0.00,120.90) 0.1793	0.02(0.00,1419.30) 0.4891	1.09 (0.97, 1.23) 0.1640
Male	1.05 (0.99, 1.11) 0.1197	0.97 (0.89, 1.04) 0.3917	0.25 (0.00, 5365.30) 0.7843	0.00 (0.00, 95.21) 0.1906	1.09 (0.98, 1.22) 0.1232
P for interaction	0.897	0.3023	0.3239	0.5627	0.9882
Dissection location					
Extracranial	1.07 (1.01, 1.12) 0.0115	0.96 (0.91, 1.01) 0.1308	0.00 (0.00, 2.04) 0.0679	0.00 (0.00, 7.56) 0.1374	1.09 (0.99, 1.19) 0.0662
Intercranial	0.94 (0.78, 1.12) 0.4628	0.97 (0.80, 1.17) 0.7302	0.00 (0.00, inf) 0.6188	0.59 (0.00, inf) 0.9623	1.13 (0.90, 1.42) 0.2881
P for interaction	0.1369	0.9587	0.9725	0.6577	0.7663
Hypertension					
No	1.03 (0.97, 1.10) 0.3406	1.02 (0.94, 1.11) 0.6219	0.00(0.00, 24663.58) 0.4195	16.64 (0.00, inf) 0.6987	0.99 (0.90, 1.09) 0.8268
Yes	1.04 (0.98, 1.10) 0.1729	0.91 (0.84, 0.99) 0.0375	0.02 (0.00, 308.46) 0.4162	0.00 (0.00, 1.47) 0.0574	1.11 (0.97, 1.27) 0.1204
P for interaction	0.8129	0.0595	0.7627	0.1154	0.1594
Hyperlipidemia					
No	1.05 (1.00, 1.09) 0.0304	0.97 (0.92, 1.02) 0.2194	0.00 (0.00, 6.67) 0.1254	0.01 (0.00, 17.62) 0.2252	1.08 (0.99, 1.17) 0.0772
Yes	305950.92(0.00,inf)0.9922	72.31 (0.00, inf) 0.9922	inf (0.00, inf) 0.9922	0.00 (0.00, inf) 0.9922	0.16 (0.00, inf) 0.9922
P for interaction	0.1033	0.0831	0.0682	0.2235	0.0425
DM					
No	1.04 (1.00, 1.09) 0.0389	0.97 (0.92, 1.02) 0.1933	0.00 (0.00, 7.81) 0.1324	0.00 (0.00, 9.42) 0.1682	1.06 (0.99, 1.15) 0.1032
Yes	470.58 (0.00, inf) 0.9922	26009.72(0.00,inf)0.992	0.00 (0.00, inf) 0.9922	0.00 (0.00, inf) 0.9922	inf (0.00, inf) 0.9922
P for interaction	0.1105	0.0899	0.135	0.1036	0.0966
Morphology subtypes					
Pearl-and-string appearance	0.98 (0.81, 1.18) 0.8160	0.93 (0.74, 1.17) 0.5446	0.00 (0.00, inf.) 0.3641	0.08 (0.00, inf.) 0.8290	1.13 (0.93, 1.37) 0.2321
Steno-occlusion only	1.01 (0.96, 1.06) 0.6444	0.96 (0.89, 1.03) 0.2105	0.00 (0.00, 4.19) 0.0793	0.00 (0.00, 48.54) 0.1929	1.05 (0.94, 1.18) 0.3723
Aneurysmal dilatation only	1.11 (1.00, 1.22) 0.0518	0.99 (0.92, 1.06) 0.6925	252,448.73(0.01,inf)0.1461	0.50(0.00,1,059,079.75)0.925	1.04 (0.93, 1.17) 0.4981
P for interaction	0.1732	0.7688	0.0359	0.7386	0.7851
VAH					
None	1.05 (1.00, 1.10) 0.0446	0.97 (0.92, 1.02) 0.2662	0.00 (0.00, 13.05) 0.1425	0.00 (0.00, 15.86) 0.1916	1.07 (0.98, 1.17) 0.1469
Ipsilateral	1.06 (0.94, 1.20) 0.3632	0.94 (0.80, 1.11) 0.4823	0.03(0.00,388,166.7)0.663	0.00(0.00,164,068.5)0.469	0.90 (0.66, 1.23) 0.5002
Opposite	0.89 (0.61, 1.30) 0.5614	1.00 (0.84, 1.18) 0.9689	0.03 (0.00, inf) 0.8443	inf (0.00, inf) 0.6018	1.21 (0.80, 1.82) 0.3706
Bilateral	1.00 (0.00, inf) 1.0000	1.00 (0.00, inf.) 1.0000	1.00 (0.00, inf) 1.0000	1.00 (0.00, inf) 1.0000	1.00 (0.00, inf) 1.0000
P for interaction	0.7328	0.9703	0.9820	0.8038	0.5653

^§^variables were expressed as OR (95% CI) *P* value

^*^Statistically significant at *P* < 0.05

*DM* diabetes mellitus, *VAH* vertebral artery hypoplasia, *OR* odds ratio, *CI* confidence intervals

## Discussion

sVAD is undoubtedly a critical etiology of posterior ischemic stroke. However, relying solely on clinical characteristics to predict IS occurrence in sVAD patients may be insufficient. Evidence suggests that vascular morphological features play a pivotal role in the outcome and progression of arterial dissection diseases. Despite this, there remains a lack of systematic analysis and precise quantification of these morphological features in sVAD patients [12, 29, 30]. Identifying significant morphologic features could facilitate early detection of a high-risk probability of IS in sVAD patients, thereby assisting in establishing more effective clinical strategies. Furthermore, it may be invaluable for clinicians to conveniently monitor sVAD patients at a high-risk probability of IS. Our study utilized 3D reconstruction techniques, offering enhanced accuracy in identifying morphological variants for sVAD progression. We developed a novel prediction model to assess the IS risk in sVAD patients, incorporating five morphologic parameters (BA length, V4 length, curvature of the V2, curvature of the V3, VA ostium angle) and five clinical features (sex, dissection location, hypertension, VAH). The AUC of the prediction model was 0.944 (95%CI, 0.862–0.984) in the training cohort and 0.818 (95%CI, 0.597–0.948) in the validation cohort, demonstrating a high accuracy and reliability. To the best of our knowledge, this study strengthens the evidence that VBS morphological features significantly impact the progression of sVAD.

In the current study, the occurrence percentage of IS (80.65%) aligns with previous findings. Interestingly, we found that VA ostium angle was smaller in the IS group (75.97° ± 14.77) compared with the non-IS group (84.88° ± 15.36) and VA ostium angle had the highest AUC among morphologic predictors. Furthermore, VBO angle less than 78.22° was prone to progress to IS in our study. In fact, the origin of VA has been described as diverse [17]. However, since all patients in our study exhibited type 1 or 2 aortic arch, we defined the VAO angle as the angle between the proximal VA and the subclavian artery (SCA) (Fig. 1B). Essentially, variation of artery bifurcation angle is correlated to the alterations of FSS and hemodynamic environment [31, 32]. A previous studies found that more severe ISR had a larger angle of VAO [33]. In contrast, our study demonstrated that VAO angle was smaller in the sVAD with IS group than the non-IS group. Several possible reasons could elucidate this phenomenon. On the one hand, fluid shear stress (FSS) is proven to be a dominant factor of hemodynamics, playing a critical role in maintaining the structure of arterial vessels. Wang et al. detected that the maximum wall shear stress (WSS) experienced by artery is positively related to the bifurcation angle [34]. In detail, as the bifurcation angel increases, the region of low FSS decreases. This reduction in low FSS has a protective impact on artery wall, as low FSS can induce inflammatory-cell-mediated destructive remodeling [35]. Besides, another CFD study of VBO has demonstrated that the area of low FSS was decreases with the VBO angle changing from 74.72° to 80.53°, while the area of low FSS was positively associated with the VBO angle during the range of 80.53° to 95.37° [36]. Specifically, this study found that an angle of 74.72° manifested the highest area of low FSS, indicating a higher likelihood of thrombosis development. In our study, the optimal threshold of the VBO angle for predicting IS event was 78.22°, which was close to this angle corresponding to a high thrombosis risk [37, ]. On the other hand, a narrow VBO angle implies a more direct blood flow into the VA, potentially inducing an adverse hemodynamic environment [36, 38]. And it is noteworthy that a larger inflow angle appears more prone to across thrombosis [37, 39 There is still controversial in determining the optimal VBO angle to avoid abnormal FSS and blood flow patterns in vertebral artery diseases across prior studies. Thus, comprehending the hemodynamic alterations and mechanisms associated with the changes in the VBO angle is vital for improving disease management and risk stratification. Future research will continue to validate the optimal VBO angle cut-off for assessing IS risk and its potential hemodynamics mechanisms in sVAD patients.

Apart from VBO angle, BA length and V4 length were also significant predictors of IS risk. IS patients were more prone to have a shorter BA length and a longer V4 length compared to non-IS patients in our study. These two morphological parameters are essential components of VBS. A previous laboratory study, based on postmortem humans specimens, revealed detailed the unique hemodynamic environment in the VBS and provided a strong impetus for further investigation [40]. Evaluating flow features of VBS can be beneficial for studying the development and initiation of VBS diseases [41]. This is why our study has shed light on the morphological characteristics of the VBS. In line with previous research, BA length emerged as a risk factor for IS occurrence in sVAD patients [42]. The negative correlation of BA length with IS occurrence in sVAD patients may be attributed to the BA angle increasing as the BA length decreases (Figure S1). A narrow BA angle, implying more pronounced BA bending, was correlated with a greater risk of posterior ischemic stroke [43]. Nevertheless, the differences in BA curvature and tortuosity between the IS and non-IS groups were not statistically significant. This may be due to the limited sample size or the potential offsetting of their impacts by other more crucial risk factors in our study.

The V4 segment was longer in sVAD patients with IS in our study. This could be explained by the positive correlation between V4 length and V4 tortuosity observed in our study (Figure S1). Importantly, several studies have reported that arterial torture not only elevates the susceptibility to acute ischemic stroke [44], but also deteriorates the severity of cerebral white matter hyperintensities in IS patients [45]. Owing to the structural variations and complexities within the VBS [46], there is no consensus on dominant morphological variables associated with vascular diseases in the VBS. The knowledge of these morphological features in the VBS could assist clinicians in developing effective strategies for the treatment of sVAD. However, our results on VBS features warrant further validation.

In addition to tortuosity, VA curvature was also a crucial parameter in our current study, aligning with previous literature [13]. Specifically, curvature of the V2 segment and curvature of the V3 segment were included in our final model. In fact, arterial curvature should not be neglected while evaluating hemodynamics in vascular diseases [47], owing to more vortical blood flow of attributable to secondary flow in a curved pipe than in a straight pipe [48]. Another experimental research also observed that cell adhesion of circulating blood was more pronounced in curved vessels than in straight vessels, suggesting a higher probability of platelet adhesion in curved vessels, thereby impacting thrombus formation [49]. Consequently, it is understandable that more curved VAs may be more prone to ischemic events. Besides, sVAD patients who are male, presence of hypertension, intracranial dissection, presence of VAH were found to have a greater risk of ischemic stroke in our study, which is in agreement with previous studies [6–9, 14, 50, 51]. Altogether, the rigorous quantification and systematic measurement of vessel morphometrics especially the bifurcation angle, in patients with sVAD, represent one of the strengths of our study.

However, we also acknowledge some limitations of the present study. First, there is an inevitable selective and recall bias due to its retrospective design. Second, we only enrolled unilateral and isolated sVAD patients. Although this stringent and rigorous inclusion criteria benefit in controlling the confounders such as multiple and bilateral dissections, this relatively modest sample size might constrain the extension of our findings. Nevertheless, we performed LASSO regression to avoid over-adjustment, a common approach when the number of variables greatly exceeds the sample size. Moreover, we utilized tenfold cross-validation and bootstrap resampling to address dataset imbalances. Third, although this model was well validated in the internal validation cohort, further external validation will be necessary in the future. This can be attributed to the complexity of 3D reconstruction and morphology measurement, making it challenging to extend to subcenters. Fortunately, we are engaged in the design and development of a more convenient and reliable automatic artificial intelligence (AI) software, enabling validation of our findings in a larger sample cohort by applying this AI tool in future studies.

In conclusion, we developed a reliable and clinically useful prediction model incorporating vessel morphometric features and clinical characteristics by applying 3D reconstruction and automatic morphological measurement. Specifically, a narrow VSO angle, short BA length, extended V4 length, increased V2 curvature, and increased V2 curvature might increase individual vulnerability to IS in sVAD patients. Our findings suggest that VBS anatomical variants play a pivotal role in the risk stratification of sVAD. Future studies are needed to validate these morphological features and explore their underlying hemodynamic mechanisms.

## Supplementary Information

Below is the link to the electronic supplementary material.

Supplementary Material 1.

## Data Availability

The data and material in this study are available from the corresponding author upon reasonable request.
