# Supplementary material for: Morphological Features of the Vertebrobasilar System Predict Ischemic Stroke Risk in Spontaneous Vertebral Artery Dissection
Source: J Cardiovasc Transl Res. 2024 Jul 9;17(6):1365–76. doi: 10.1007/s12265-024-10534-6 (PMC11634921; doi:10.1007/s12265-024-10534-6)
Supplement: Supplementary file 1 — Supplementary Material 1. [file 12265_2024_10534_MOESM1_ESM.docx]

**Table S1**. Optimal Cut-off Values of Morphological Parameters.

|  |  | VA ostium angle | V4 length | V2 curvature | V3 curvature | BA length |
| --- | --- | --- | --- | --- | --- | --- |
| Best threshold | | 78.22 | 49.6024 | 0.1916 | 0.1876 | 28.5479 |
| Specificity | | 0.53 | 0.5067 | 0.3067 | 0.40 | 0.7867 |
| Sensitivity | | 0.83 | 0.8333 | 0.9444 | 0.8333 | 0.50 |
| Accuracy | | 0.59 | 0.5699 | 0.4301 | 0.4839 | 0.7312 |
| Positive-LR | | 1.79 | 1.6892 | 1.3622 | 1.3889 | 2.3438 |
| Negative-LR | | 0.31 | 0.3289 | 0.1812 | 0.4167 | 0.6356 |
| Diagnose-OR | | 5.71 | 5.1351 | 7.5192 | 3.3333 | 3.6875 |
| N-for-diagnose | | 2.73 | 2.9412 | 3.9823 | 4.2857 | 3.4884 |
| Positive-pv | | 0.30 | 0.2885 | 0.2464 | 0.25 | 0.36 |
| Negative-pv | | 0.93 | 0.9268 | 0.9583 | 0.9091 | 0.8676 |


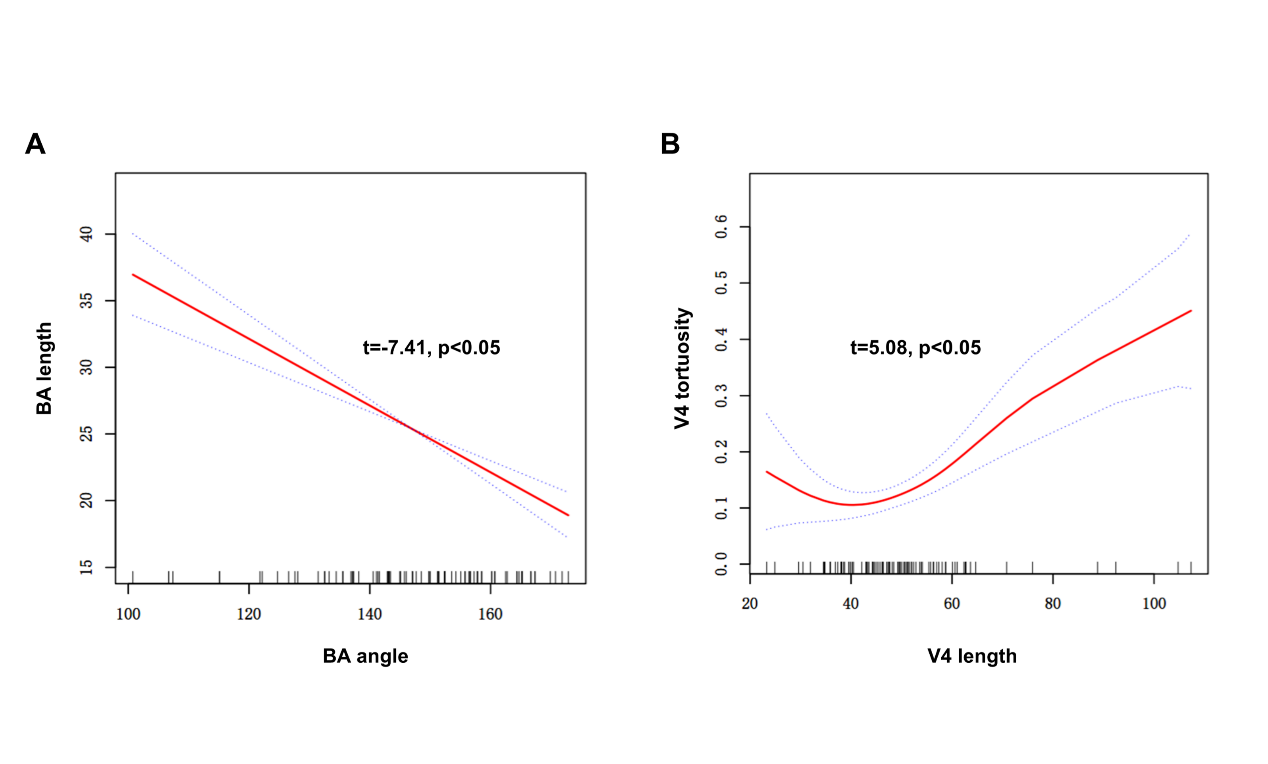


**Fig** S1. A: correlation between the basilar artery length and basilar artery angle. B: correlation between the V4 tortuosity and V4 length.
